# New methodologies at PF AR-NW12A: the implementation of high-pressure macromolecular crystallography

**DOI:** 10.1107/S0909049513020797

**Published:** 2013-10-01

**Authors:** Leonard Michel Gabriel Chavas, Tadayuki Nagae, Hiroyuki Yamada, Nobuhisa Watanabe, Yusuke Yamada, Masahiko Hiraki, Naohiro Matsugaki

**Affiliations:** a Structural Biology Research Center, PF/IMSS/KEK, 1-1 Oho, Tsukuba, Ibaraki 300-0801, Japan; b Venture Business Laboratory, Nagoya University, Nagoya, Aichi 464-8603, Japan; c Department of Biotechnology, Graduate School of Engineering, Nagoya University, Nagoya, Aichi 464-8603, Japan; d Synchrotron Radiation Research Center, Nagoya University, Furo-cho Chikusa-ku, Nagoya, Aichi 464-8603, Japan

**Keywords:** HPMX, DAC, high pressure, PDIS

## Abstract

The macromolecular crystallography (MX) beamline AR-NW12A is evolving from its original design of high-throughput crystallography to a multi-purpose end-station. Among the various options to be implemented, great efforts were made in making available high-pressure MX (HPMX) at the beamline. High-pressure molecular biophysics is a developing field that attracts the interest of a constantly growing scientific community. A plethora of activities can benefit from high pressure, and investigations have been performed on its applicability to study multimeric complex assemblies, compressibility of proteins and their crystals, macromolecules originating from extremophiles, or even the trapping of higher-energy conformers for molecules of biological interest. Recent studies using HPMX showed structural hydrostatic-pressure-induced changes in proteins. The conformational modifications could explain the enzymatic mechanism differences between proteins of the same family, living at different environmental pressures, as well as the initial steps in the pressure-denaturation process that have been attributed to water penetration into the protein interior. To facilitate further HPMX, while allowing access to various individualized set-ups and experiments, the AR-NW12A sample environment has been revisited. Altogether, the newly added implementations will bring a fresh breath of life to AR-NW12A and allow the MX community to experiment in a larger set of fields related to structural biology.

## Abstract 2

The evolution of AR-NW12A into a multi-purpose end-station with optional high-pressure crystallography is described.

## 1. Introduction

The Photon Factory Advanced Ring (PF-AR) will soon enter its 30th year of operation, following its initial commissioning in 1984. The PF-AR is a 6.5 GeV ring that operates in a single-bunch mode, with two injections per 24 h run. Among the eight beamlines opened for user applications at PF-AR, two end-stations for macromolecular crystallography (MX) are currently in use, namely AR-NE3A (Yamada *et al.*, 2010 ▶) on the Northeast wing and AR-NW12A (Chavas *et al.*, 2012 ▶) on the Northwest wing of the ring. AR-NW12A recently celebrated its ten-year anniversary as a high-throughput MX beamline, making it the oldest available MX end-station at the Photon Factory, with over 650 publications and 700 coordinates deposited in the Protein Data Bank (http://www.rcsb.org). The beamline’s work has spanned two Japanese national structural biology projects, that is, the *Protein 3000* project and the *Targeted Proteins Research Program* project. As Japan begins its third national project [*Platform for Drug Discovery, Informatics, and Structural Life Science* or PDIS (http://www.pford.jp)], it will be upgraded to a multipurpose experimental beamline, with the addition of a variety of equipment aimed at facilitating new experimental approaches, including high-pressure macromolecular crystallography (HPMX), high-pressure freezing of macromolecular crystals, *in situ* UV/Vis microspectrometry, and *in situ* plate screening.

HPMX is a powerful tool that allows pressures up to several hundred megapascals to be reached to facilitate the accessibility to different conformational states of protein molecules. Pressure has long been applied to study biochemical systems in detail, while keeping the temperature and volume effects separate. HPMX has been successfully used for the study of multimeric complex assemblies (Girard *et al.*, 2010 ▶), crystal compressibility (Ascone *et al.*, 2010 ▶), the trapping of higher-energy conformers (Collins *et al.*, 2005 ▶), and for the analysis of macromolecules originating from extremophiles. We recently investigated the structural differences between proteins of the same family living at different environmental pressures, and identified conformational modifications in 3-isopropylmalate dehydrogenase induced by hydrostatic pressure, with water penetration into the protein interior that initiated a pressure-denaturation process (Nagae *et al.*, 2012 ▶).

The present communication intends to describe the implementation of HPMX at AR-NW12A, with details of the various equipment and future developments to be incorporated during the upgrade of the beamline. The addition of HPMX tools to the current diffractometer is necessary for allowing general users, unfamiliar with this technique, to test their samples and utilize the full potential of this approach, as access to HPMX-only end-stations remains sporadic. Within such a schema, we strongly hope to further attract the user community and to contribute to the exponential growth of interest in HPMX.

## 2. Instrumentation

### 2.1. Pressure-cell design

By definition, the pressure applied to an object corresponds to the amount of force applied on the object’s surface per unit area. In HPMX, the experimental pressure limit that can be exercised on a macromolecular crystal is restricted by the sensitivity of the crystal to keep its long-range ordering or uniformity of the proteins inside. The higher pressure that can be generated by the pressure cell will, therefore, have a direct influence on the limitations of the experiments. In practice, however, the sample cavity in which the protein crystal lies is filled with a liquid (usually the crystal mother liquor) that will determine hydrostatic compression on the crystal. Therefore, the maximum pressure that can be applied to a macromolecular system in HPMX experiments will be limited by the phase diagram of the hydrostatic liquid, with an upper limit of roughly 1 GPa for water at 293 K, and 2 GPa at 350 K. Such pressures only influence the intramolecular distances and eventually modify conformations, keeping covalent bond distances and angles constant. Accordingly, the maximum pressure reported in the literature for HPMX studies was 1 GPa, for bovine erythrocyte Cu,Zn superoxide dismutase (Ascone *et al.*, 2010 ▶).

**Figure 1:**
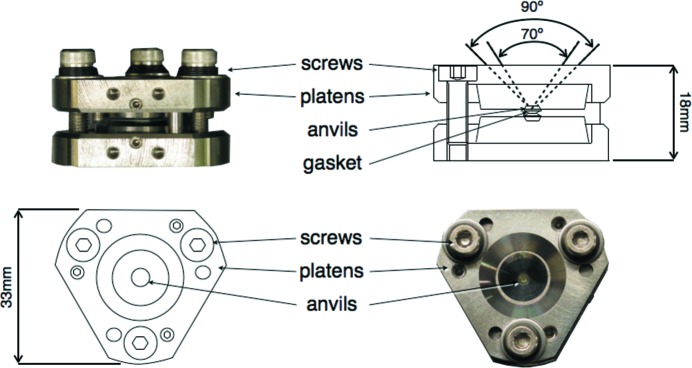
Diagrams and photographs of the Merrill and Bassett type DAC available at AR-NW12A. The data-collection aperture angle (70°) and the diamond anvil optical aperture angles (90°) are indicated by dotted lines, viewed from the side of the DAC.

A large variety of pressure cells for X-ray studies are commercially available, each cell being specifically designed for a particular application. The blind cylindrical cells of the Kundrot & Richards (1986 ▶) type were used in early HPMX studies. These pressure cells are made of beryllium, which allowed collection of data for a wide range of diffraction angles, but with a serious restriction in pressure at 200 MPa. The difficulty in visualizing the samples through the beryllium cell represented an additional drawback of this approach. Diamond-anvil cells (DACs) designed for X-ray experiments by Merrill & Bassett (1974 ▶) were first used for compressibility experiments on lysozyme crystals in 1996 (Katrusiak & Dauter, 1996 ▶). The principles of the DACs and the generation of high pressure within can be found in numerous reviews (Fourme *et al.*, 2001 ▶). The design of the DAC presently available at AR-NW12A shows a wide optical aperture for collecting data over a large rotation angle, up to approximately 70° (Fig. 1 ▶). This key feature helps in collecting high-completeness data, which is especially important for crystals with low-symmetry space groups. Additionally, the compactness of the cell (33 and 18 mm for the longest and shortest dimensions, respectively) enables straightforward implementation in the multi-purpose sample diffractometer, without interfering with other components. The DAC cullet has a diameter of 1 mm, surrounding the diamond anvils that sandwich a gasket of quenched stainless steel with an aperture of 600–700 µm and a thickness of 300 µm. The large sample chamber, defined by the gasket wall and the two diamond anvils, accommodates several crystals for a single set of experiments, suitable for alleviating the fast degradation induced by X-ray damage when irradiating biological samples at room temperature.

### 2.2. Sample alignment unit

The sample alignment unit consists of the combination of a goniometer, an observation camera and a backlight. For standard experiments, AR-NW12A is equipped with a single-axis horizontal goniometer with an interchangeable gonio­meter-head that adapts to a variety of sample holders. Although its low cylinder of confusion (2 µm; Kohzu Precision) permits precise positioning of crystals within cryo-loops at the beam position, the uncertainty in centring the samples increases proportionally with the weight of the sample holder itself (∼200 g for the DAC and holder, compared with ∼1 g for conventional MX sample holders). To improve the sphere of confusion when working on small crystals, a vertical gonio­meter is now being designed. It will be implemented in addition to the presently available horizontal goniometer. We are making a significant effort to prepare a multi-faceted system that will permit the co-existence of both mounting devices with fast exchangeability of the set-up.

**Figure 2:**
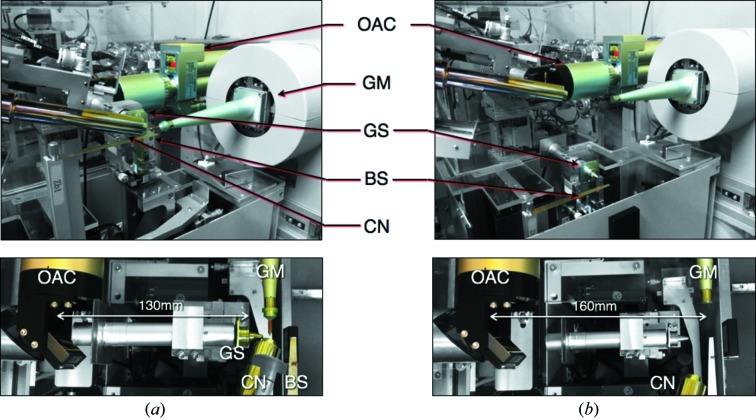
The diffractometer set-up for (*a*) cryo-loop *versus* (*b*) HPMX experiments. Images in the bottom panels are viewed from the top of the diffractometer. OAC, on-axis camera; GM, goniometer; GS, guard scattering; BS, beam stopper; CN, cryo-stream nozzle.

Samples are visualized in the direction of the X-ray beam through an on-axis camera (Zoom Microscope; Sigma Koki). Because biological crystals cannot be fixed inside the DAC, they are highly susceptible to moving inside the sample chamber, while the cell rotates during data collection. The evident advantage of working with an on-axis visualization system lies in the fact that what is observed optically is what the incoming X-rays will encounter, thus reducing the uncertainties in sample manipulation. The focus point of the implemented camera is located at approximately 160 mm from a 45°-inclined mirror, which opens a large space for DAC manipulation (Fig. 2 ▶). The optical magnification of the telescope (7×) together with the visual resolution of the camera (e-line up to 4.2 µm) allows unambiguous resolution of small objects. A backlight powered at 150 W illuminates the sample. Its brightness and relative position can be adjusted through the beamline control software to facilitate clear visualization of the samples.

### 2.3. Diffractometer

As described previously (Chavas *et al.*, 2012 ▶), most of the devices related to the experimental environment of the crystals are regrouped near the sample position and are retractable on demand. For general applications, sample crystals are normally mounted in cryo-loops and tightly surrounded by a guard slit (GS), a cryo-stream nozzle (CN) and a direct beam stopper (BS) within a few millimetres (Fig. 2 ▶). For HPMX experiments, the GS and BS remain at a distance from the pressure cell, to avoid collision during sample rotation. The gasket of the DAC replaces the GS, while a rear BS implemented near the CCD detector substitutes the front BS. Because the effects of temperature and pressure on the internal free energy of molecules are of comparable amplitude, both variables should be kept constant during an experiment. Hence, the distance of the CN from the sample position and the temperature of the cryo-stream jet can be freely modified through the beamline control software.

## 3. Methods

### 3.1. Pressure calculations

The variation of pressure favours the bi-directional, uniform and quick change of the volume of a system. As in all high-pressure studies, a reliable method should be available for measuring precisely the pressure applied to the studied sample. The calculation of pressure strongly depends on the pressure type to be measured. In HPMX experiments, the pressure is derived from the changes of the volume enclosed within a cavity inside the gasket. As a consequence, measurement of the pressure has to be indirect. By following the change of energy spectrum in the sharp-line (R-line) luminescence of ruby (Forman *et al.*, 1972 ▶), the pressure within a closed volume can be measured accurately. For HPMX at AR-NW12A, the R-line spectrum of a pink ruby crystal enclosed inside the DAC is measured offline on a helium–neon laser (Showa Optics). One obvious drawback of a Merrill and Bassett type DAC lies in the difficulties in automating pressure generation, as pressure is mechanically generated by pulling screws together. Moreover, the measure of pressure on the offline system requires removing the DAC from the gonio­meter every time a pressure change is to be recorded. To limit this burden, an on-line fluorescence measurement system should be installed together with the installation of the vertical goniometer.

### 3.2. Choice of X-ray energy and beam size

**Figure 3:**
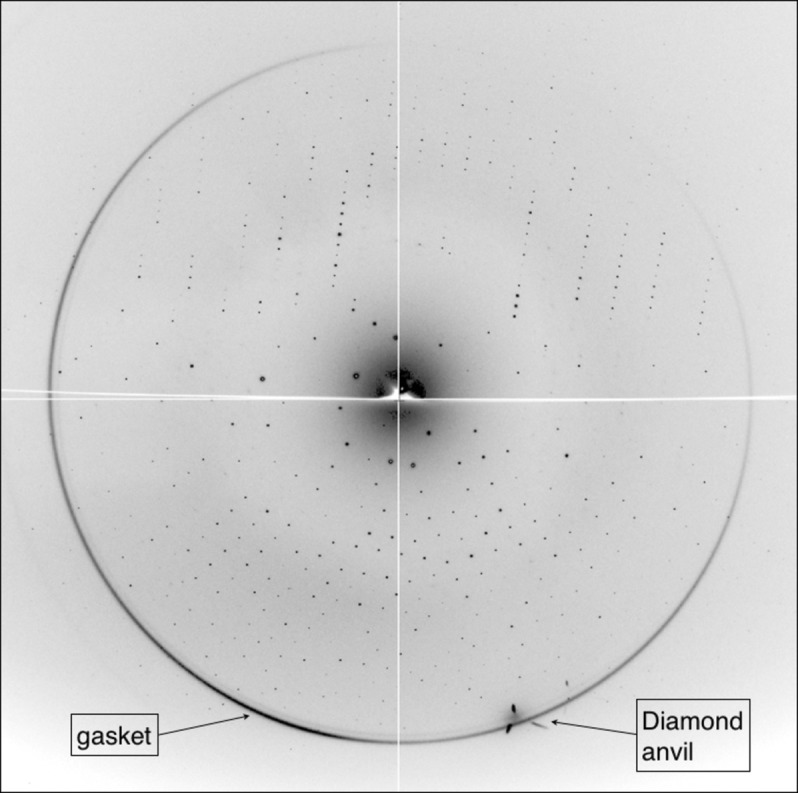
Typical diffraction pattern from a protein crystal submitted to high pressure. The present data were recorded at a sample-to-detector distance of 200 mm, which gives a diffraction limit of 1.2 Å at the edge of the detector. The diamonds from the anvil are emitting scarce Bragg spots with a pyramidal shape at around 2.0 Å, which are clearly differentiated from the gasket scattering (also at 2.0 Å) and from the protein Bragg spots.

When measuring high-quality diffraction data for HPMX experiments, care should be provided in selecting the optimum wavelength. The chosen energy will have a direct influence on the resolution, signal-to-noise ratio, X-ray absorption by cell components, and will be dependent on the detector characteristics, often optimized for a specific set of energies. Ultra-short wavelengths of the order of 0.25–0.05 Å have been reported as being optimal for HPMX studies (Fourme *et al.*, 2001 ▶). At AR-NW12A, the shortest wavelength available is 0.70 Å, limited by the cut-off (∼3.5 mrad) of the focusing mirror reflectivity downstream of the monochromator, rather than by the X-ray undulator source (see Fig. 1 of Chavas *et al.*, 2013 ▶). A typical diffraction pattern recorded during HPMX experiments is shown Fig. 3 ▶. Although sub-optimal, data collection at 0.70 Å should increase the crystal lifetime approximately by a factor of two, when compared with data collection at 1.00 Å, in accordance with calculations of the dose-dependent decay of crystals exposed to wavelengths of 0.70 Å and 1.00 Å, respectively, with a conserved incoming flux of 1.0 × 10^11^ photons s^−1^ mm^−2^ in an area of 200 µm × 200 µm (calculations performed using *Raddose* version 2.0; Paithankar *et al.*, 2009 ▶). Moreover, the ADSC Quantum 210r CCD X-ray detector implemented at AR-NW12A is calibrated to have its detective quantum efficiency (DQE) maximized at the wavelength of 1 Å. Although no major troubles could be observed in the data recorded so far with an energy beam of 17.7 keV (0.70 Å), HPMX at AR-NW12A could improve in data quality if a detector calibrated for higher energies was available.

The recommended beam size at AR-NW12A varies from 100 µm × 100 µm to 200 µm × 200 µm, in positive cases where access to sufficiently large crystals is possible. A clear advantage of this large beam size lies in the fact that a larger volume of the crystal is exposed to the X-ray beam, resulting in increased signal at higher energies. A comparison of data collected at PF AR-NW12A with data recorded at SPring-8 BL41XU (Kawamoto *et al.*, 2001 ▶), where a sharper beam was available, clearly suggested that a larger beam size coupled with a lower flux was beneficial for collecting fully usable data at room temperature (unpublished results).

## 4. Targeted areas of research

Owing to its intrinsic thermodynamic and kinetic properties, HPMX is a powerful tool for understanding the regulation of bio-events. Since the first HPMX experiments performed at AR-NW12A, interest in this technique began to emerge. The implementation of tools for HPMX at the end-station has favoured studies of the pressure deactivation processes of several proteins, in close relationship with their biological functions (Nagae *et al.*, 2012 ▶). The importance of water molecules in the deactivation events was highlighted, and additional work has been performed in this direction. In previous works, Nagae and colleagues describe how water penetration in the interior of proteins originating from the deep seas might explain increased enzyme activity at high pressures, as studied at AR-NW12A (Nagae *et al.*, 2012 ▶). Additionally, conformational changes of proteins were highlighted with changes in the space group while growing in pressure (Yamada *et al.*, unpublished data). It is worth noting that industrial applications in specific biotechnological areas, such as high-pressure food processing, are being considered; there the use of HPMX would be highly beneficial. Finally, one additional field of interest is detailed studies of cold de­naturation (Privalov, 1990 ▶). During this study of the denaturation process of globular proteins at low temperatures, the use of HPMX to shift the freezing point of aqueous protein solutions would allow accessing sub-zero temperatures without ice. The direct influence of sub-zero temperatures on the stability of proteins could then be addressed.

## 5. Conclusions

Until recently, the complexity of combining X-ray protein crystallography and high-pressure approaches was reflected in the scarcity of HPMX studies at the few available X-ray sources. There has been a large increase of interest by the macromolecular crystallographic community in HPMX experiments. Although the AR-NW12A beamline is still at its initial phase of upgrading to a multi-purpose end-station, the success of HPMX is an encouraging result. To facilitate further HPMX, while maintaining accessibility for various atypical set-ups and experiments, the AR-NW12A sample environment is being revisited, and various improvements will be made in the near future. The newly added equipment will bring a new breath of life to AR-NW12A, allowing the MX community to experiment with a larger set of parameters relevant to biological systems.

## References

[bb1] Ascone, I., Kahn, R., Girard, E., Prangé, T., Dhaussy, A.-C., Mezouar, M., Ponikwicki, N. & Fourme, R. (2010). *J. Appl. Cryst.* **43**, 407–416.

[bb2] Chavas, L. M. G., Matsugaki, N., Yamada, Y., Hiraki, M., Igarashi, N., Suzuki, M. & Wakatsuki, S. (2012). *J. Synchrotron Rad.* **19**, 450–454. 10.1107/S0909049512009727 22514184

[bb3] Chavas, L. M. G., Yamada, Y., Hiraki, M., Igarashi, N., Matsugaki, N. & Wakatsuki, S. (2013). *J. Phys. Conf. Ser.* **425**, 012008.

[bb4] Collins, M. D., Hummer, G., Quillin, M. L., Matthews, B. W. & Gruner, S. M. (2005). *Proc. Natl Acad. Sci. USA*, **102**, 16668–16671. 10.1073/pnas.0508224102 PMC1283839 16269539

[bb5] Forman, R. A., Piermarini, G. J., Barnett, J. D. & Block, S. (1972). *Science*, **176**, 284–285. 10.1126/science.176.4032.284 17791916

[bb6] Fourme, R., Kahn, R., Mezouar, M., Girard, E., Hoerentrup, C., Prangé, T. & Ascone, I. (2001). *J. Synchrotron Rad.* **8**, 1149–1156. 10.1107/s0909049501011037 11524565

[bb7] Girard, E., Marchal, S., Perez, J., Finet, S., Kahn, R., Fourme, R., Marassio, G., Dhaussy, A. C., Prangé, T., Giffard, M., Dulin, F., Bonneté, F., Lange, R., Abraini, J. H., Mezouar, M. & Colloc’h, N. (2010). *Biophys. J.* **98**, 2365–2373. 10.1016/j.bpj.2010.01.058 PMC2872268 20483346

[bb8] Katrusiak, A. & Dauter, Z. (1996). *Acta Cryst.* D**52**, 607–608. 10.1107/S0907444996000431 15299694

[bb9] Kawamoto, M., Kawano, Y. & Kamiya, N. (2001). *Nucl. Instrum. Methods Phys. Res. A*, **467**, 1375–1379.

[bb10] Kundrot, C. E. & Richards, F. M. (1986). *J. Appl. Cryst.* **19**, 208–213.

[bb11] Merrill, L. & Bassett, W. A. (1974). *Rev. Sci. Instrum.* **45**, 290–294.

[bb12] Nagae, T., Kawamura, T., Chavas, L. M. G., Niwa, K., Hasegawa, M., Kato, C. & Watanabe, N. (2012). *Acta Cryst.* D**68**, 300–309. 10.1107/S0907444912001862 PMC3282623 22349232

[bb13] Paithankar, K. S., Owen, R. L. & Garman, E. F. (2009). *J. Synchrotron Rad.* **16**, 152–162. 10.1107/S0909049508040430 19240327

[bb14] Privalov, P. L. (1990). *Crit. Rev. Biochem. Mol. Biol.* **25**, 281–305. 10.3109/10409239009090612 2225910

[bb15] Yamada, Y., Hiraki, M., Sasajima, K., Matsugaki, N., Igarashi, N., Amano, Y., Warizaya, M., Sakashita, H., Kikuchi, T., Mori, T., Toyoshima, A., Kishimoto, S. & Wakatsuki, S. (2010). *AIP Conf. Proc.* **1234**, 415–418.

